# Metagenomic analysis of the *Rhinopithecus bieti* fecal microbiome reveals a broad diversity of bacterial and glycoside hydrolase profiles related to lignocellulose degradation

**DOI:** 10.1186/s12864-015-1378-7

**Published:** 2015-03-12

**Authors:** Bo Xu, Weijiang Xu, Junjun Li, Liming Dai, Caiyun Xiong, Xianghua Tang, Yunjuan Yang, Yuelin Mu, Junpei Zhou, Junmei Ding, Qian Wu, Zunxi Huang

**Affiliations:** School of Life Science, Yunnan Normal University, Kunming, 650500 China; Engineering Research Center of Sustainable Development and Utilization of Biomass Energy, Ministry of Education, Kunming, 650500 China; Key Laboratory of Yunnan for Biomass Energy and Biotechnology of Environment, Kunming, 650500 China; Key Laboratory of Enzyme Engineering, Yunnan Normal University, Kunming, 650500 China

**Keywords:** Gastrointestinal microbiota, *Rhinopithecus bieti*, Metagenomics, Lignocellulose degradation, Pyrosequencing

## Abstract

**Background:**

The animal gastrointestinal tract contains a complex community of microbes, whose composition ultimately reflects the co-evolution of microorganisms with their animal host and the diet adopted by the host. Although the importance of gut microbiota of humans has been well demonstrated, there is a paucity of research regarding non-human primates (NHPs), especially herbivorous NHPs.

**Results:**

In this study, an analysis of 97,942 pyrosequencing reads generated from *Rhinopithecus bieti* fecal DNA extracts was performed to help better understanding of the microbial diversity and functional capacity of the *R. bieti* gut microbiome. The taxonomic analysis of the metagenomic reads indicated that *R. bieti* fecal microbiomes were dominated by Firmicutes, Bacteroidetes, Proteobacteria and Actinobacteria phyla. The comparative analysis of taxonomic classification revealed that the metagenome of *R. bieti* was characterized by an overrepresentation of bacteria of phylum Fibrobacteres and Spirochaetes as compared with other animals. Primary functional categories were associated mainly with protein, carbohydrates, amino acids, DNA and RNA metabolism, cofactors, cell wall and capsule and membrane transport. Comparing glycoside hydrolase profiles of *R. bieti* with those of other animal revealed that the *R. bieti* microbiome was most closely related to cow rumen.

**Conclusions:**

Metagenomic and functional analysis demonstrated that *R. bieti* possesses a broad diversity of bacteria and numerous glycoside hydrolases responsible for lignocellulosic biomass degradation which might reflect the adaptations associated with a diet rich in fibrous matter. These results would contribute to the limited body of NHPs metagenome studies and provide a unique genetic resource of plant cell wall degrading microbial enzymes. However, future studies on the metagenome sequencing of *R. bieti* regarding the effects of age, genetics, diet and environment on the composition and activity of the metagenomes are required.

**Electronic supplementary material:**

The online version of this article (doi:10.1186/s12864-015-1378-7) contains supplementary material, which is available to authorized users.

## Background

The Yunnan snub-nosed monkey (*Rhinopithecus bieti*) is an endangered colobine endemic to high-altitude forests ranging from 3000 to 4400 meters in southwestern China and southeastern Tibet [1]. Overall, these colobines can be classified as herbivores, ingesting flowers, fruits, leaves, and seeds to varying degrees [2]. The *R. bieti* possesses specialized S-shaped and partitioned stomachs where microbial fermentation of cellulose takes place [3,4]. This adaptation enables them to eat food containing high levels of structural polysaccharides, i.e., cellulose and related compounds. Given the above features, this species has received significant attention from researchers and serves as an important model organism for studying the evolution of the primate diet. However, researches on *R. bieti* have mostly focused on aspects of taxonomy, ecology, anatomy and conservation genetics.

The gastrointestinal tract of animals harbors a complex microbial community, and the composition of this community ultimately reflects the co-evolution of microorganisms with their animal host and the diet adopted by the host [5]. It’s well known that herbivores lack the enzymatic capacity needed to degrade plant polysaccharides, particularly cellulose, and instead rely on community of microorganisms that have this capacity. Therefore, *R. bieti* are expected to have a well-adapted gut microbiota. Indeed, the limited studies published to date suggest the microbiomes of *R. bieti* possess a large number of bacteria that may be involved in degradation of cellulose [6]; however, very little is currently known about the genetic potential and structure-function relationships intrinsic to these microbiomes.

Recently, next-generation sequencing technologies have been used to characterize the microbial diversity and functional capacity of a range of microbial communities in the gastrointestinal tracts of humans [7-10] as well as in several animal species [11-25]. The most important advantages of this cloning-independent approach are the avoidance of cloning bias and the bias introduced by PCR amplification. To the best of our knowledge, this study was the first to apply a random sample pyrosequencing approach to analyze the metagenome of *R. bieti*, an herbivore whose habitat and diet are very different compared to the herbivores studied so far.

## Results and discussion

The analysis of the reads yielded a high percentage of species identification in complex metagenomes and even higher in less complex samples. Long sequence reads from 454 GS FLX Titanium pyrosequencing provided the high specificity needed to compare the sequenced reads with the DNA or protein databases and allowed the unambiguous assignment of closely related species. The initial pyrosequencing runs yielded 97,942 reads containing 37,482,416 bases of sequence, with an average read length of 382 bp. Prior to further processing, the raw read data were subjected to the Metagenome Rapid Annotation using Subsystem Technology (MG-RAST) v.3.0 online server quality control pipeline [26] to remove duplicate and low quality reads (Additional file 1). The filtering step removed 9.6% of reads in the sample. The unique sequence reads that passed the quality control (QC) filtering step were then subjected to further analysis focusing on biodiversity and functional annotation. All reads were deposited in the National Center for Biotechnology Information (NCBI) and can be accessed in the Short Read Archive (SRA) under the accession number SRX493843.

### Phylogenetic analysis of *R. bieti* fecal bacteria, eukaryota, archaea, and viruses

**Figure 1:**
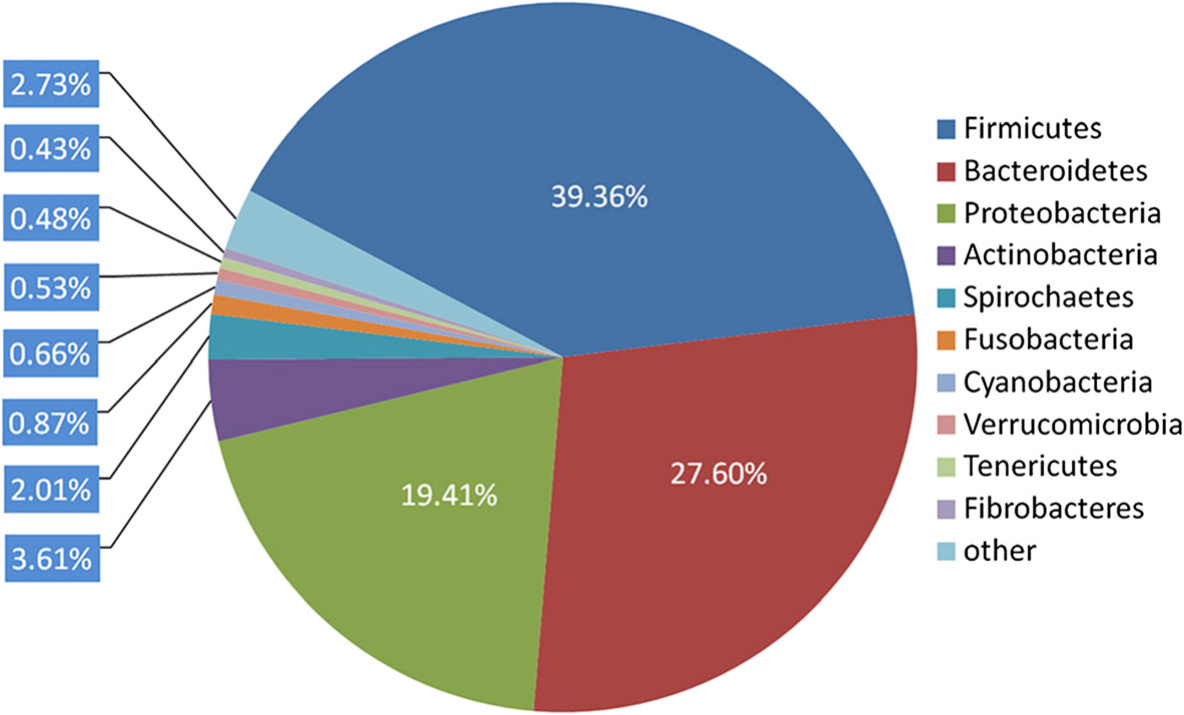
**Bacterial phylum profiles of the** ***R. bieti*** **microbiome.** The percentage of the *R. bieti* fecal metagenomic sequences assigned to M5NR database is shown. Through the “Organism Abundance” tool in MG-RAST, the *R. bieti* fecal sequencing runs were determined from the M5NR database with the BLASTx algorithm. The e-value cutoff for the metagenomic sequence matches to the M5NR database was 1 × 10^−5^, with a minimum alignment length of 30 bp.

The overview of the phylogenetic computations provided 97.81% bacteria, 1.27% eukaryota, 0.73% archaea, and 0.17% viruses. In the *R. bieti* intestinal metagenome, Firmicutes was the most predominant phylum (39.36%), followed by Bacteroidetes (27.6%), Proteobacteria (19.41%), Actinobacteria (3.61%) and Spirochaetes (2.01%) (Figure 1). Compared with the previous 16S rRNA gene-based data [6], Firmicutes were the dominant bacteria phylum. In addition, higher percentages of Bacteroidetes and lower percentages of Spirochaetes in the *R. bieti* intestinal metagenome were observed. This discrepancy may have been caused by the biases associated with the primers, PCR reaction conditions, or selection of clones [27].

Within the Firmicutes group, Clostridiales were most predominant, among which *Clostridium* was overrepresented (Additional file 2), which is consistent with previous 16S rRNA gene-based data [6]. The presence of a large proportion of *Clostridia* was likely to be important for lignocellulosic biomass degradation [28]. Several cellulolytic microbes such as *C. phytofermentans*, *Ruminococcus albus, C. thermocellum*, *C. cellulolyticum*, *R. flavefaciens* and *C. cellulovorans* were abundant in the *R. bieti* metagenome (Additional file 2). Among clostridial genomes sequenced to date, *C. phytofermentans* has the highest number of genes encoding enzymes for the modification and breakdown of complex carbohydrates. It contains genes for 161 carbohydrate-active enzymes (CAZy), which include 108 glycoside hydrolases (GH) spreading across 39 families [29]. *C. thermocellum* is an anaerobic thermophilic bacterium that exhibits one of the highest rates of cellulose utilization among described microorganisms [28]. *R. flavefaciens* is a specialist cellulolytic bacterial species characterized in the rumen, other herbivorous animals and humans. Currently it is the only rumen bacterium known to produce a defined cellulosome [30] which is usually associated with improved cellulolytic efficiency [31]. These cellulolytic *Clostridia*, which are ubiquitous in cellulosic anaerobic environments, represent a major paradigm for efficient biological degradation of cellulosic biomass [32].

Bacteroidetes were the second predominant phylum in the *R. bieti* gastrointestinal tract with Bacteroidales as the primary contributor to the Bacteroidetes populations, followed by Flavobacteria, Sphingobacteriales and Cytophagales. The major genus in the Bacteroidetes phylum was *Bacteroides. Bacteroides* are commonly found in the human intestine where they have a symbiotic host-bacterial relationship with humans. They assist in breaking down food and producing valuable nutrients and energy that the body needs. *B. vulgatus*, *B. fragilis* and *Flavobacterium johnsoniae* comprise about 1.1%, 1.1%, and 1.08% of the reads analyzed respectively (Additional file 2); therefore, it is considered the predominant species in the *R. bieti* metagenome. *B. fragilis* is a ubiquitous Gram-negative anaerobic bacterium that inhabits the lower gastrointestinal tract of most mammals [5]. Recent findings have revealed that this organism possesses the ability to direct the cellular and physical maturation of the host immune system and protect its host from experimental colitis [33-35]. *B. vulgatus* is among the most commonly isolated microbes from the human gastrointestinal tract, and it has been found to constitute part of the core gut microbiota in healthy humans [10,36]. According to the CAZy classification scheme, *B. vulgatus* is the only sequenced gut Bacteroidetes with a gene encoding a xylanase [37]. *F. johnsoniae* digests many polysaccharides and proteins, but it is best known for its ability to rapidly digest insoluble chitin [38]. *F. johnsoniae* and other members of the Bacteroidetes phylum are thought to play important roles in the turnover of this compound in many environments [39]. Perhaps the major metabolic function of these dominant intestinal bacteria is the fermentation of nondigestible carbohydrates including large polysaccharides (i.e., pectins and cellulose), which are key sources of energy in the *R. bieti* colon.

Similarly, Burkholderiales were the primary contributors to the Proteobacteria populations, followed by Enterobacteriales and Pseudomonadales. The major genus in the Proteobacteria phylum was *Pseudomonas. P. fluorescens* was the predominant species among the *Pseudomonas* in the *R. bieti* metagenome. *P. fluorescens* is a common Gram-negative bacterium that can be found in the low section of the human digestive tract [40].

**Figure 2:**
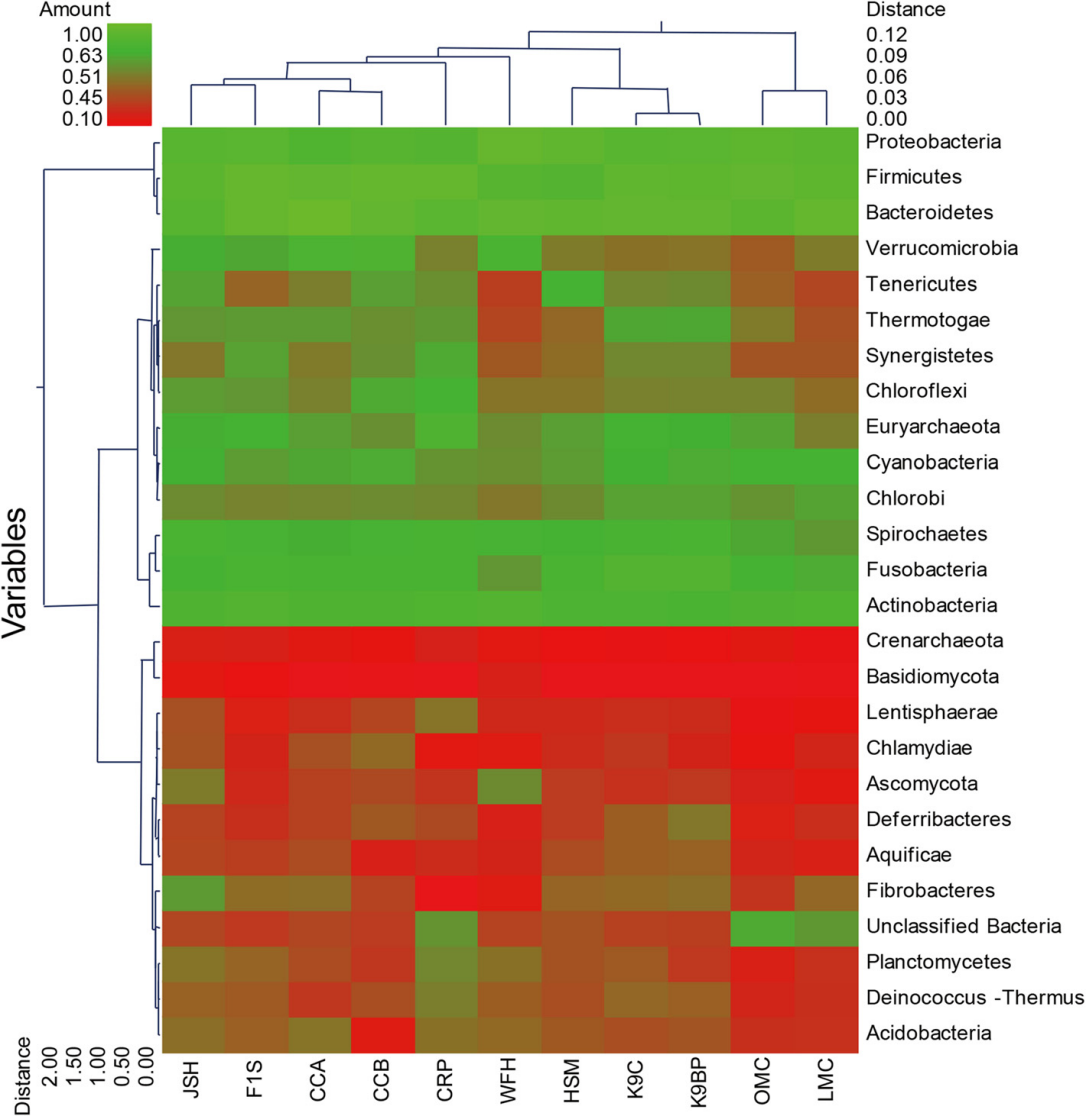
**Phylogenetic clustering of** ***R. bieti*** **, pygmy loris, human, mouse, canine, cow, and chicken gastrointestinal metagenomes.** A double hierarchical dendrogram was established through weight-pair group clustering methods based on the non-scaling Manhattan distance. The dendrogram shows the phylogenetic distribution of the microorganisms among the eleven metagenomes from the seven different hosts, including *R. bieti* (JSH), pygmy loris (WFH), human (HSM and F1S), mouse (LMC and OMC), dog (K9C and K9BP), cow (CRP), and chicken (CCA and CCB). The linkages of the dendrogram do not show the phylogenetic relationship of the bacterial phylum and are based on the relative abundance of taxonomic profiles. The heat map depicts the relative percentage of each phylum of microorganism (variables clustering on the y axis) in each sample (x axis clustering). The heat map color represents the relative percentage of the microbial descriptions in each sample, with the legend indicated at the upper left corner. Branch length indicates the Manhattan distances of the samples along the x axis (scale at the upper right corner) and of the microbial phyla along the y axis (scale at the lower left corner).

A distinctive feature of the *R. bieti* metagenome is the abundance of phylum Fibrobacteres and Spirochaetes, and this abundance is unexpected and far greater than in other animals (Figure 2). *Fibrobacter succinogenes* was the only species existed in the *R. bieti* gut (Additional file 2) that is recognised as a major bacterial degrader of lignocellulosic material in the herbivore gut [41]. It was originally thought that members of the genus *Fibrobacter* were restricted to the mammalian intestinal tract, but the occurrence and distribution of members of the Fibrobacteres phylum has recently been extended to include termite intestinal contents where cellulose is again the primary carbon source for the host organisms [42]. Spirochaetes form a distinct monophyletic phylum of bacteria, and contain four genera that contain important pathogenic species, these being *Treponema*, *Borrelia*, *Leptospira* and *Brachyspira*, which were all exist in the *R. bieti* metagenome (Additional file 2). Morphologically diverse Spirochaetes are consistently present in the hindgut of all termites [43], and are found out as ectosymbionts attached to the surface of cellulose-digesting protists [44].

Eukaryota were a minor constituent (1.27%) in the *R. bieti* metagenome. Species of *Blastocystis* which have been reported as the most commonly occurring micro-eukaryote in human feces [45,46] were also represented in small quantities (<0.01%) in the *R. bieti* metagenome. In addition, the presence of *Blastocystis* has been linked to a number of gut-related diseases. Some of these diseases could be the outcome of the predation of beneficial bacteria by *Blastocystis* in light of the similar observations in ruminant cattle and their communalistic protozoa [47].

Fungi have very low abundance sequences (0.26%), with Ascomycota being the primary contributor (Additional file 3). Compared with humans, more diverse fungi species belonging to 21 different genera exist in the *R. bieti* metagenome. The most abundant fungi genera in the *R. bieti* metagenome were *Aspergillus* (0.06%), *Gibberella* (0.03%), *Magnaporthe* (0.02%) and *Neurospora* (0.02%) (Additional file 3). Fungi in the intestinal ecosystem of NHPs have not yet been studied extensively. Comparative analyses indicate that both fungal populations in the *R. bieti* and pygmy loris metagenomes [25] were predominated by *Aspergillus*. However, the most abundant fungi species within *Aspergillus* of the *R. bieti* metagenome, *A. fumigatus*, has not been detected in pygmy loris. *A. fumigatus* is one of the most common human pathogen*.* The whole genome sequence of *A. fumigatus* shows that up to 18 different genes encoding endoglucanases have been annotated, which indicates that this species own a good capacity in lignocellulose hydrolysis [48]. Moreover, two fungi species (*Neurospora crassa* and *Gibberella zeae*) have been identified predominantly both in the *R. bieti* (Additional file 3) and pygmy loris metagenome [25], which were also identified in feline [19], canine (K9C and K9BP) [16], and mouse metagenomes (OMC) [49].

Unlike other NHPs, *R. bieti* are herbivorous mammals that have a specialized gut in which plant materials degraded by microbial processes apparently similar to those that occur in the rumen. In rumen ecosystems, fungi interact with other microbes to take part in decomposing cellulose. Thus, it seems likely that anaerobic fungi may play significant role in fiber degradation in the *R. bieti*. Future studies are required by next-generation sequencing to gain further insight into the fungal diversity in the *R. bieti* gastrointestinal tract.

Archaea are a minor component of the *R. bieti* metagenome, comprising 0.73% of the total sequencing reads. Archaea consist of two phyla, Euryarchaeota and Crenarchaeota, which diverged into night classes and eleven orders (Additional file 4). Among the groups of archaea, methanogenic archaea is the most predominant and diverse group. Methanogenic archaea is also widespread in chicken, dogs, felines, mice, ruminants, NHPs and humans [12,16,19,25,49-51]. In the *R. bieti* fecal metagenome, *Methanocorpusculum labreanum* is the major component of archaea, having a percentage of 0.05% in all the analytic sequences (Additional file 4), which is consistent with pygmy loris [25]. The majority of the Archaea in the rumen are methanogens which provide thermodynamically favorable conditions for ruminal microbial fibre degradation [52]. Although methanogenic archaea make up only a small part of the *R. bieti* microbial population, they may play an important role in microbial fermentation like in the rumen system. Archaea are considered commensals; however, they contribute to pathogeny in humans because of mutual interactions with other microorganisms [53]. For instance, methanogens consume hydrogen and create an environment that enhances the growth of polysaccharide fermenting bacteria, leading to higher energy utilization. Higher numbers of methanogenic archaea have been observed in obese humans [54]. However, the prevalence and medical importance of archaea in *R. bieti* need to be determined.

Only 0.17% of the total reads have viral origin, with only the order Caudovirales being identified. Three families were observed (Myoviridae, Podoviridae and Siphoviridae) within the Caudovirales order, and all sequences were classified as bacteriophages (Additional file 5). Bacteriophages influence food digestion by regulating microbial communities in the human gastrointestinal tract through lytic and lysogenic replication [55]. Bacteriophages also contribute to human health by controlling invading pathogens [56]. Recent metagenomic analyses of the DNA viruses from human feces have revealed that the majority of DNA viruses in human feces are novel, and most of the recognizable sequences also belong to bacteriophages [57]. The close phylogenetic relationship between humans and NHPs, coupled with the exponential expansion of human populations and human activities within the primate habitats, has resulted in the exceptionally high possibility of pathogen exchange [58]. Therefore, studies on the viral community of NHPs and the potential for cross-transmission between humans and NHPs are needed. Given the type of methodology (shotgun DNA pyrosequencing approach) that we utilized, our study could only determine the dsDNA virus. Future studies need to provide a richer understanding of both RNA and dsDNA viruses to complete human knowledge of the viral intestinal ecosystem.

Studies on cow [59], cats [60], mice [61], rats [62] as well as humans [63] and NHPs [64] revealed a correlation between host diet and microbial community composition. However, the characterization of the dietary-induced changes in NHPs microbiomes through high-throughput sequencing technologies has not been performed thus far. Hence, more attention should be given to it in future experiments.

### Metagenomics-based metabolic profiles

**Figure 3:**
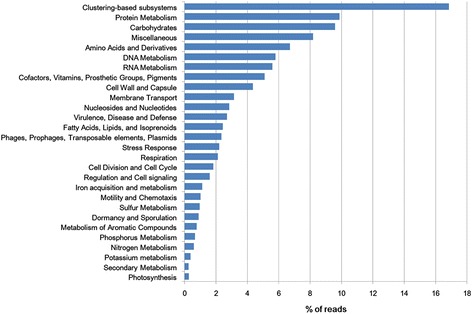
**Functional composition of the** ***R. bieti*** **microbiome.** The percentage of the *R. bieti* fecal metagenomic sequences assigned to the general SEED subsystems is shown. Through the “Functional Abundance” tool in MG-RAST, the *R. bieti* fecal sequencing runs were determined from the SEED database with the BLASTx algorithm. The e-value cutoff for the metagenomic sequence matches to the SEED subsystem database was 1 × 10^−5^ with a minimum alignment length of 30 bp.

Protein and carbohydrate metabolism are the most abundant functional categories, representing 9.87% and 9.59% of the *R. bieti* fecal metagenomes respectively (Figure 3). Genes associated with amino acids and derivatives, DNA metabolism, RNA metabolism, cofactors (vitamins, prosthetic groups, pigments), cell wall and capsule and membrane transport are also very abundant in the *R. bieti* metagenomes. Approximately 16.85% of the annotated reads from the *R. bieti* fecal metagenomes were categorized within the clustering-based subsystems, most of which have unknown or putative functions.

Compared with NHPs pygmy loris gastrointestinal microbiomes [25], protein metabolism was more enriched in the gut microbiomes of *R. bieti*. On further analysis, it seemed that *R. bieti* had enriched protein biosynthesis in which universal GTPases were the most abundant. Because *R. bieti* eat foods rich in fibre, it is likely *R. bieti* have more available substrate for bacterial fermentation. This increase in protein biosynthesis may simply be the result of higher metabolic activity and/or growth of microbial populations.

### Diversity of fibrolytic enzymes in the *R. bieti* metagenome

To obtain a more in-depth view of the carbohydrate enzymes present in *R. bieti* fecal microbiome, we subjected our samples to the CAZy database (http://www.cazy.org), as described by Cantarel et al. [29]. The comparison of the 88,514 metagenome reads post-QC processing based on the CAZy database provided 2,315 hits at an e-value restriction of 1 × 10^−6^. Candidate sequences that belong to the glycosyl transferase (GT) families GT2 (268) are the most abundant, followed by members of the GT4 (189) and glycoside hydrolase (GH) families GH13 (165) (Additional files 6 and 7).

In the *R. bieti* fecal metagenomes there is a wide diversity of GH catalytic modules with >1,300 sequences belonging to 79 GH families. GHs are a prominent group of enzymes that hydrolyze the glycosidic bond among the carbohydrate molecules. The most frequently occurring GH families in the *R. bieti* metagenome were GH13 (165; 12.61% of the total GH matches), 2 (106; 8.1%), 3 (83; 6.35%) and 43 (61; 4.66%) (Additional files 6 and 7). Family GH13 is the major glycoside hydrolase family acting on substrates containing α-glucoside linkages. The majority of the enzymes acting on starch, glycogen, and related oligo- and polysaccharides, are found within family GH13, which represents the largest family of glycoside hydrolases. In adition to α-amylases, it contains pullulanase, cyclomaltodextrin glucanotransferase (CGTase), cyclomaltodextrinase, neopullulanase, α-glucosidase, etc. (www.cazy.org). Some members of family GH13 bearing a variable number of supplemental N- or C-terminal extensions such as starch-binding modules (carbohydrate-binding modules (CBM) 26, CBM41 and CBM34 in CAZy) [65] were all annotated in the *R. bieti* metagenome (Additional file 7).

Families GH2 and GH3, which contain a large range of glycosidases cleaving nonreducing carbohydrates in oligosaccharides and the side chains of hemicelluloses and pectins, were abundant less than GH13. GH2 components are β-D-galactosidases, β-glucuronidases, β-D-mannosidases, and exo-β-glucosaminidases. The most common activities of GH3 include β-D-glucosidases, α-L-arabinofuranosidases, β-D-xylopyranosidases, and N-acetyl-β-D-glucosaminidases [66]. GH43 shows β-xylosidase, β-1,3-xylosidase, α-L-arabinofuranosidase, arabinanase, xylanase, and galactan 1,3-β-galactosidase activity (www.cazy.org). Glycosyl transferases are ubiquitous enzymes that catalyze the attachment of sugars to a glycone [67]. Candidate genes that belong to the glycosyl transferase families GT2 (268; 36.12% of the total GT matches) and GT4 (189; 25.47%) are the most abundant (Additional file 6).

Comparative analysis of GH families was done between metagenomes of the *R. bieti*, human, Tammar, termite hindgut, and cow rumen. Clustering analysis of the GH family distribution implied that the *R. bieti* metagenome was most closely related to cow rumen (Additional file 8). The GH5 were the most abundant cellulases in all metagenomes, which occurred at the highest frequency in the termite metagenome. Similar to the termite [11] and rumen [15] metagenomes, *R. bieti* was more evenly balanced with GH5 and GH9 cellulases. However, GH9 cellulases occurred at a lower frequency in human [10] metagenomes and were not even detected in the macropod [14] metagenomic datasets. GH28 hemicellulases were prevalent in the *R. bieti* microbiome; however, they occurred at a lower frequency in other animal gut microbiomes. Debranching enzymes profile in the *R. bieti* microbiome was similar to those reported for wallaby foregut microbiome [14]. The *R. bieti* microbiome possessed a large number of reads matching GH family specific for oligosaccharides, in which the most abundant oligosacchride-degrading enzymes were GH2, GH3, and GH43. Although both *R. bieti* and humans belong to primates, the GH profiles targeting plant structural polysaccharides in the two metagenomes are not more similar than other animals. This could be the result of the diet differences. Overall, the distribution of GH family enzymes in the micobiomes of *R. bieti* generally reflected its adaptation to special food types.

### Comparative metagenomic analysis

Despite the extensive variation among individuals, the gut microbiota of members of the same species are often more similar to one another compared with those of other species. Thus, it is important to provide a comparison between the gastrointestinal microbiomes of primates and those of other animals. The results of this study were compared with data sets from different animals and even humans in the MG-RAST database. Paired data from other studies were chosen, such as lean (LMC) and obese (OMC) mouse cecal metagenomes [49], two chicken cecal metagenomes (CCA, CCB) [12], two canine intestinal metagenomes (K9C, K9BP) [16], and two human fecal metagenomes (F1S, HSM). F1S was a healthy human fecal metagenome [51], whereas HSM was defined as human feces from a malnourished subject. In addition,a cow rumen and a NHPs pygmy loris [25] fecal metagenomes were also utilized for comparison. The comprehensive overview of the data sets is shown in Additional file 9.

**Figure 4:**
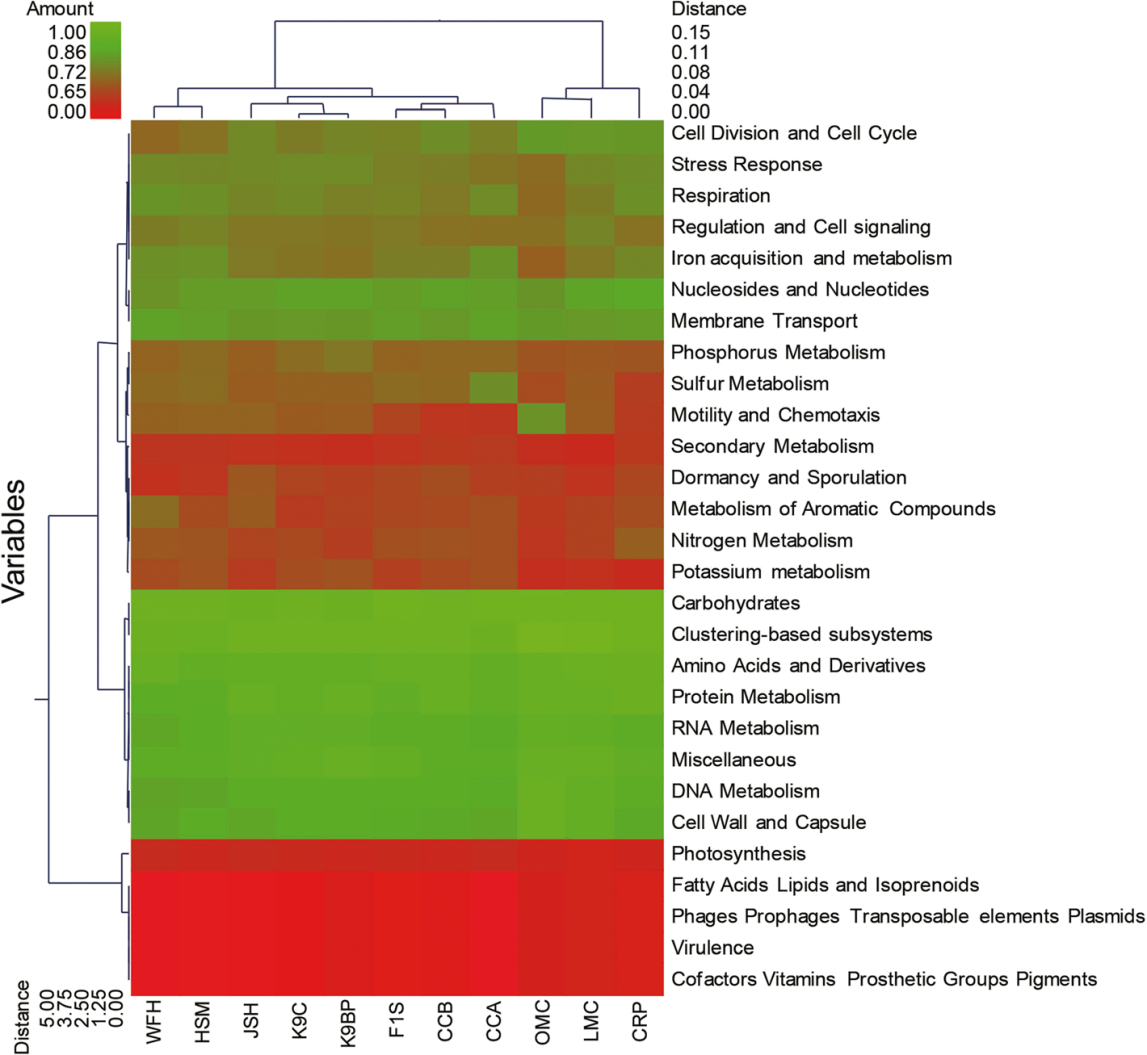
**Metabolic clustering of** ***R. bieti*** **, pygmy loris, human, mouse, canine, cow, and chicken gastrointestinal metagenomes.** A double hierarchical dendrogram was established through a weight-pair group clustering method based on the non-scaling Manhattan distance. The dendrogram shows the distribution of the functional categories among the eleven metagenomes from the seven different hosts, including *R. bieti* (JSH), pygmy loris (WFH), humans (HSM and F1S), murine (LMC and OMC), canine (K9C and K9BP), cow (CRP), and chicken (CCA and CCB). The linkages of the dendrogram are based on the relative abundance of metabolic profiles. The heat map depicts the relative percentage of each category of function (variables clustering on the y axis) in each sample (x axis clustering). The heat map color represents the relative percentage of functional categories in each sample, with the legend indicated at the upper left corner. Branch length indicates the Manhattan distances of the samples along the x axis (scale at the upper right corner) and of the microbial classes along the y axis (scale at the lower left corner).

Clustering the metagenomes was carried out with unscaled Manhattan variance distances and presented through a double hierarchical dendrogram. The clustering-based comparisons were demonstrated at the phylogenetic level (Figure 2) and the metabolic level (Figure 4). In the phylogenetic comparison, the *R. bieti* samples clustered with the human fecal metagenomes (F1S), two chicken cecal metagenomes (CCA and CCB), a cow rumen metagenome and a pygmy loris fecal metagenome and separated from those of the two mouse metagenomes (OMC and LMC). Non-metric multidimensional scaling (MDS) plot illustrated that the distances among the *R. bieti* and cow rumen as well as among HSM, K9C and K9BP were the nearest (Additional file 10). This may be due to similar bacterial diversity influenced by similar diet rich in fibre within *R. bieti* and cow. In all the samples, the Actinobacteria, Bacteroidetes, Firmicutes and Proteobacteria were the most abundant (Figure 2). The heat map also demonstrates that the *R. bieti* metagenome was most distinguished by the greater prevalence of Fibrobacteres, an important phylum of cellulose-degrading bacteria, compared with other animals.

Metabolism-based hierarchical clustering demonstrates that the *R. bieti*, dog, chicken, human and pygmy loris samples clustered together and separated from those of the mice and cow (Figure 4). Non-metric MDS plot illustrated that all metagenomics data have more than 80% similarity (Additional file 11). As expected, all the gut metagenomes were dominated by carbohydrate metabolism subsystems with amino acids, protein, and cell wall and capsule; the DNA and RNA subsystems were represented in relatively high abundance as well (Figure 4).

## Conclusions

We presented for the first-time the application of the shotgun metagenomic pyrosequencing approach to study the fecal microbiome of the *R. bieti*. The overall goal of this study was to characterize the species composition and the functional capacity of the *R. bieti* fecal microbiome. Taxonomic analysis of the metagenomic reads showed similarities among the gut microbiomes of the *R. bieti*, humans and other animals. Four phyla dominated the microbiomes, namely, Actinobacteria, Bacteroidetes, Firmicutes and Proteobacteria. However, the relative proportion of the phyla was different. At the genus-level taxonomic resolution, *Bacteroides* species were the most abundant, most of which were represented by *B. vulgates* and *B. fragilis*. The organisms belonging to the same genus also represent one of the most abundant microbial taxa in the human intestinal microbiota [7,68]. The *R. bieti* faecal samples contained more bacteria belonging to the phylum Fibrobacteres, all of which were represented by the lignocellulose-degrading bacterium *F. succinogenes*. The high amount of *F. succinogenes* present in the *R. bieti* feces indicates a high turnover of lignocellulose in this NHPs. Archaea, fungi, and viruses are minor constituents of the *R. bieti* fecal microbiome. All archaea are members of Crenarchaeota and Euryarchaeota, with methanogens being the most abundant and diverse. Two fungi phylotypes were present in the *R. bieti* fecal microbiome, namely, Ascomycota and Basidiomycota, with Ascomycota being the primary contributor. Only about 0.17% of sequences were of viral origin, and all sequences were classified as bacteriophages. Overall, the microbial populations of the *R. bieti* gut system appeared to consist of taxa with known capacities for degradation and utilizing foods high in fibrous matter.

The comparative metagenomic analyses identified unique and/or overabundant taxonomic and functional elements in the *R. bieti* distal gut microbiomes. Relatively abundant bacteria of phylum Fibrobacteres and Spirochaetes were found in the *R. bieti* metagenome compared with all the other gut metagenomes. Primary functional categories were similar to those of other gut microbiomes and were associated mainly with protein, carbohydrates, amino acids, DNA and RNA metabolism, cofactors (vitamins, prosthetic groups, pigments), cell wall and capsule and membrane transport. Comparing GH profiles of *R. bieti* with those of herbivores, we found that the *R. bieti* microbiome possessed a large number of GH family specific for oligosaccharides, similar to that for the cow rumen. These findings may reflect the evolutionary adaptations for the highly specialized herbivory of *R. bieti*.

These results contribute to the limited body of primate metagenome studies and provide a unique genetic resource of plant cell wall degrading microbial enzymes. More studies involving the deeper sequencing of metagenomes are required to fully characterize the gastrointestinal microbiome of the *R. bieti* and other NHPs in healthy and diseased states, of varying ages or genetic backgrounds, and in the wild or in captivity.

## Methods

### Fecal sample collection

Fresh fecal samples from eight *R. bieti* were collected at a single time point from the Baimaxueshan National Nature Reserve of Weixi, Yunnan Province, China, with the permission of the authorities of Baimaxueshan National Nature Reserve. We tracked the *R. bieti* until they defecated; the fecal samples were immediately collected aseptically. The fresh fecal samples were transported to the laboratory on dry ice within 24 hours of collection, and then stored at −80°C until DNA extraction. We brought no toxic substance that would have adverse effects on the biotic community to minimize disturbance in the animal habitats. The research complied with the protocols established by the China Wildlife Conservation Association and adhered to the American Society of Primatologists (ASP) Principles for the Ethical Treatment of Non-Human Primates as well as the legal requirements of China.

### DNA extraction and shotgun pyrosequencing

Genomic DNA were extracted from the fecal samples with the QIAamp DNA stool mini kit (Qiagen, Valencia, CA, USA) following the protocol provided by the supplier (0.25 g of each fecal sample). The quality and quantity of the DNA were determined with a nanodrop (ND-1000) spectrophotometer (Nanodrop Technologies, Wilmington, DE, USA) through agarose gel electrophoresis. DNA samples were stored frozen (−20°C) until use.

A total of 500 ng of pooled DNA was subjected to library preparation and shotgun pyrosequencing using the Roche 454 GS FLX Titanium System (Roche, Basel, Switzerland) as it was not feasible to distinguish which fecal sample corresponded to which individual. The obtained reads were uploaded to MG-RAST [26] under the name JSH_Metagenome and were assigned the Metagenome ID: 4452795.3. The MG-RAST v.3.0 online server quality control pipeline was utilized to remove reads of short length and poor quality before annotation and the analysis of metagenomic data [26]. The pipeline parameters were kept at default settings.

### Bioinformatics and statistical analysis

Comparative metagenomic analysis was performed with MG-RAST pipelines. The metagenomic runs from the *R. bieti* data were compared with the current publicly available gut metagenomes in the databases. In the MG-RAST metagenomic annotation pipeline, the *R. bieti* fecal metagenomic datasets were compared with ten public sets of data from animals, including chicken cecum A (CCA 4440283), chicken cecum B (CCB 4440284), two dog metagenome data sets (K9C 4444164 and K9BP 4444165), lean mouse cecum (LMC 4440463), obese mouse cecum (OMC 4440464), cow rumen (CRP 4441682), human stool metagenome (HSM 4444130), human F1-S feces metagenome (F1S 4440939) and pygmy loris fecal metagenome (WFH 4476304). The organisms in MG-RAST were classified through the M5NR protein database (http://tools.metagenomics.anl.gov/m5nr/). The functional annotation and classification relied on the SEED subsystem ([69]; http://www.theseed.org/wiki/ Home_of_the_SEED) databases. The maximum e-value of 1e-5, minimum percent identity of 60, and minimum alignment length of 30 were applied as the parameter settings in the analysis. The taxonomic and functional profiles were normalized to determine the differences in the sequencing coverage by calculating the percent distribution prior to downstream statistical analysis. Clustering was performed using Ward’s minimum variance with unscaled Manhattan distances [70]. Heat maps were drawn by hierarchal clustering performed with NCSS 2007 (Kaysville, Utah). Non-metric MDS analysis based on Bray-Curtis’ similarity and Euclidean distance were performed using PRIMER 6 statistical software (PRIMER-E Ltd., Plymouth Marine Laboratory, Plymouth, U.K.).

Annotations based on the carbohydrate-active enzymes database ([29]; http://www.cazy.org) were provided for all the reads that passed the MG-RAST QC filter. Sequences are subject to Blast analysis against a library composed of modules derived from CAZy at an e-value restriction of 1 × 10^−6^. The cluster analysis of metagenomes on the basis of GH profile was carried out using the PAST v.2.17b data analysis package [71].

## Additional files

Additional file 1:
**Information regarding the sequence datas.**
Additional file 2:
**Phylogenetic classification of bacteria in the** ***R. bieti*** **metagenome.**
Additional file 3:
**Phylogenetic classification of fungi in the** ***R. bieti*** **metagenome.**
Additional file 4:
**Phylogenetic classification of archaea in the** ***R. bieti*** **metagenome.**
Additional file 5:
**Phylogenetic classification of viruses in the** ***R. bieti*** **metagenome.**
Additional file 6:
**Number of occurrences for the 25 most dominant carbohydrate active enzyme families.** In total, these families contain 1557 sequences, representing 67.3% of the putative carbohydrate active enzyme family. Additional file 7:
**Presence of carbohydrate active enzyme families in the** ***R. bieti*** **metagenome.**
Additional file 8:
**GH profiles targeting plant structural polysaccharides in the** ***R. bieti*** **and other metagenomes.**
Additional file 9:
**Overview of the MG-RAST metagenomes chosen for comparison.**
Additional file 10:
**Non-metric multidimensional scaling analysis based on relative abundance of taxonomic profiles among** ***R. bieti*** **, pygmy loris, human, mouse, canine, cow, and chicken gastrointestinal metagenomes.** Samples of the same host species are indicated by the same symbol. Superimposed circles represent clusters of samples at different distance values (Euclidean distance). Additional file 11:
**Non-metric multidimensional scaling analysis based on relative abundance of metabolic profiles among** ***R. bieti*** **, pygmy loris, human, mouse, canine, cow, and chicken gastrointestinal metagenomes.** Samples of the same host species are indicated by the same symbol. Superimposed circles represent clusters of samples at different similarity values (Bray-Curtis similarity).

## Abbreviations

NHPs: Non-human primates
MG-RAST: Metagenome Rapid Annotation using Subsystem Technology
QC: Quality control
NCBI: National Center for Biotechnology Information
SRA: Short Read Archive
CAZy: Carbohydrate-Active enzymes
GH: Glycoside hydrolase
GT: Glycosyl transferase
CBM: Carbohydrate-binding modules
MDS: Multidimensional scaling
ASP: American Society of Primatologists
