# Supplementary material for: Metagenomic analysis of the Rhinopithecus bieti fecal microbiome reveals a broad diversity of bacterial and glycoside hydrolase profiles related to lignocellulose degradation
Source: BMC Genomics. 2015 Mar 12;16(1):174. doi: 10.1186/s12864-015-1378-7 (PMC4369366; doi:10.1186/s12864-015-1378-7)
Supplement: Additional file 1: — Information regarding the sequence datas. [file 12864_2015_1378_MOESM1_ESM.pdf]

## Additional file 1 Information regarding the sequence datas

| | ***R. bieti* metagenome** |
|---|---|
| **Raw data** | |
| Total # of reads | 97,942 |
| Total bp | 37,482,416 |
| Mean length (bp) | 382 ± 98 bp |
| Mean GC % | 45 ± 12 % |
| **MG-RAST Analysis** | |
| Total # of reads | 97,942 |
| Total bp | 37,482,416 |
| Mean length (bp) | 382 ± 98 bp |
| Mean GC % | 45 ± 12 % |
| Total # of reads post QC | 88,514 |
| Total bp post QC | 35,073,599 |
| Mean length post QC (bp) | 396 ± 82 bp |
| Mean GC % post QC | 45 ± 12 % |
| Processed predicted protein features | 90,920 |
| Processed predicted rRNA features | 9,679 |

QC: MG-RAST 3.0 applied quality control of the reads.
