# Supplementary material for: Metagenomic analysis of the Rhinopithecus bieti fecal microbiome reveals a broad diversity of bacterial and glycoside hydrolase profiles related to lignocellulose degradation
Source: BMC Genomics. 2015 Mar 12;16(1):174. doi: 10.1186/s12864-015-1378-7 (PMC4369366; doi:10.1186/s12864-015-1378-7)
Supplement: Additional file 3: — Phylogenetic classification of fungi in the R. bieti metagenome. [file 12864_2015_1378_MOESM3_ESM.docx]

**Additional file 3 Phylogenetic classification of fungi in the *R. bieti* metagenome**

| phylum | class | order | genus | species | # hits | JSH*  (%) |
| --- | --- | --- | --- | --- | --- | --- |
| Ascomycota | Dothideomycetes | Pleosporales | Phaeosphaeria | Phaeosphaeria nodorum | 8 | 0.01 |
|  | Eurotiomycetes | Eurotiales | Aspergillus | Aspergillus clavatus | 9 | 0.01 |
|  |  |  |  | Aspergillus flavus | 6 | 0.01 |
|  |  |  |  | Aspergillus fumigatus | 22 | 0.02 |
|  |  |  |  | Aspergillus niger | 5 | 0.01 |
|  |  |  |  | Aspergillus oryzae | 6 | 0.01 |
|  |  |  |  | Aspergillus terreus | 5 | 0.01 |
|  |  |  | Emericella | Emericella nidulans | 5 | 0.01 |
|  |  |  | Neosartorya | Neosartorya fischeri | 7 | 0.01 |
|  |  |  | Penicillium | Penicillium chrysogenum | 6 | 0.01 |
|  | Leotiomycetes | Helotiales | Botryotinia | Botryotinia fuckeliana | 13 | 0.01 |
|  |  |  | Sclerotinia | Sclerotinia sclerotiorum | 5 | 0.01 |
|  | Saccharomycetes | Saccharomycetales | Scheffersomyces | Scheffersomyces stipitis | 7 | 0.01 |
|  |  |  | Yarrowia | Yarrowia lipolytica | 9 | 0.01 |
|  |  |  | Eremothecium | Eremothecium gossypii | 5 | 0.01 |
|  |  |  | Saccharomyces | Saccharomyces cerevisiae | 10 | 0.01 |
|  |  |  | Candida | Candida tropicalis | 5 | 0.01 |
|  | Schizosaccharomycetes | Schizosaccharomycetales | Schizosaccharomyces | Schizosaccharomyces pombe | 7 | 0.01 |
|  | Sordariomycetes | Glomerellales | Verticillium | Verticillium albo-atrum | 6 | 0.01 |
|  |  | Hypocreales | Gibberella | Gibberella zeae | 26 | 0.03 |
|  |  | Magnaporthales | Magnaporthe | Magnaporthe oryzae | 14 | 0.02 |
|  |  | Sordariales | Podospora | Podospora anserina | 7 | 0.01 |
|  |  |  | Neurospora | Neurospora crassa | 19 | 0.02 |
|  |  |  | Sordaria | Sordaria macrospora | 7 | 0.01 |
| Basidiomycota | Agaricomycetes | Agaricales | Schizophyllum | Schizophyllum commune | 5 | 0.01 |
| Basidiomycota | Tremellomycetes | Tremellales | Filobasidiella | Cryptococcus neoformans | 8 | 0.01 |

* Percentage of sequences identified in metagenome of *R bieti*.
