# Supplementary material for: Metagenomic analysis of the Rhinopithecus bieti fecal microbiome reveals a broad diversity of bacterial and glycoside hydrolase profiles related to lignocellulose degradation
Source: BMC Genomics. 2015 Mar 12;16(1):174. doi: 10.1186/s12864-015-1378-7 (PMC4369366; doi:10.1186/s12864-015-1378-7)
Supplement: Additional file 4: — Phylogenetic classification of archaea in the R. bieti metagenome. [file 12864_2015_1378_MOESM4_ESM.docx]

**Additional file 4 Phylogenetic classification of archaea in the *R. bieti* metagenome**

| phylum | class | order | genus | species | JSH* (%) |
| --- | --- | --- | --- | --- | --- |
| Crenarchaeota | Thermoprotei | Desulfurococcales | Staphylothermus | Staphylothermus marinus | 0.01 |
|  |  |  | Hyperthermus | Hyperthermus butylicus | 0.01 |
|  |  | Sulfolobales | Sulfolobus | Sulfolobus solfataricus | 0.01 |
|  |  |  |  | Sulfolobus tokodaii | 0.01 |
| Euryarchaeota | Archaeoglobi | Archaeoglobales | Archaeoglobus | Archaeoglobus fulgidus | 0.01 |
|  |  |  | Ferroglobus | Ferroglobus placidus | 0.01 |
|  | Halobacteria | Halobacteriales | Haloarcula | Haloarcula marismortui | 0.01 |
|  |  |  | Halobacterium | Halobacterium salinarum | 0.01 |
|  |  |  | Haloquadratum | Haloquadratum walsbyi | 0.01 |
|  |  |  | Natrialba | Natrialba magadii | 0.01 |
|  | Methanobacteria | Methanobacteriales | Methanobrevibacter | Methanobrevibacter ruminantium | 0.02 |
|  |  |  |  | Methanobrevibacter smithii | 0.04 |
|  |  |  | Methanosphaera | Methanosphaera stadtmanae | 0.02 |
|  |  |  | Methanothermobacter | Methanothermobacter marburgensis | 0.01 |
|  |  |  |  | Methanothermobacter thermautotrophicus | 0.02 |
|  | Methanococci | Methanococcales | Methanocaldococcus | Methanocaldococcus jannaschii | 0.02 |
|  |  |  | Methanococcus | Methanococcus aeolicus | 0.01 |
|  |  |  |  | Methanococcus maripaludis | 0.04 |
|  |  |  |  | Methanococcus vannielii | 0.02 |
|  |  |  |  | Methanococcus voltae | 0.01 |
|  | Methanomicrobia | Methanomicrobiales | Methanocorpusculum | Methanocorpusculum labreanum | 0.05 |
|  |  |  | Methanoculleus | Methanoculleus marisnigri | 0.02 |
|  |  |  | Methanoplanus | Methanoplanus petrolearius | 0.01 |
|  |  |  | Methanospirillum | Methanospirillum hungatei | 0.03 |
|  |  |  | Methanoregula | Methanoregula boonei | 0.02 |
|  |  |  | Methanosphaerula | Methanosphaerula palustris | 0.01 |
|  |  | Methanosarcinales | Methanosaeta | Methanosaeta thermophila | 0.01 |
|  |  |  | Methanococcoides | Methanococcoides burtonii | 0.02 |
|  |  |  | Methanohalobium | Methanohalobium evestigatum | 0.01 |
|  |  |  | Methanohalophilus | Methanohalophilus mahii | 0.01 |
|  |  |  | Methanosarcina | Methanosarcina acetivorans | 0.04 |
|  |  |  |  | Methanosarcina barkeri | 0.04 |
|  |  |  |  | Methanosarcina mazei | 0.04 |
|  | Methanopyri | Methanopyrales | Methanopyrus | Methanopyrus kandleri | 0.01 |
|  | Thermococci | Thermococcales | Pyrococcus | Pyrococcus abyssi | 0.02 |
|  |  |  |  | Pyrococcus furiosus | 0.01 |
|  |  |  |  | Pyrococcus horikoshii | 0.01 |
|  |  |  | Thermococcus | Thermococcus barophilus | 0.01 |
|  |  |  |  | Thermococcus gammatolerans | 0.01 |
|  |  |  |  | Thermococcus kodakarensis | 0.01 |
|  |  |  |  | Thermococcus onnurineus | 0.01 |
|  | Thermoplasmata | Thermoplasmatales | Picrophilus | Picrophilus torridus | 0.01 |
|  |  |  | Thermoplasma | Thermoplasma acidophilum | 0.01 |
|  | unclassified (derived from Euryarchaeota) | unclassified (derived from Euryarchaeota) | Aciduliprofundum | Aciduliprofundum boonei | 0.02 |
|  | unclassified (derived from Euryarchaeota) | unclassified (derived from Euryarchaeota) | unclassified (derived from Euryarchaeota) | uncultured methanogenic archaeon RC-I | 0.02 |

* Percentage of sequences identified in metagenome of *R bieti*.
