# Supplementary material for: Metagenomic analysis of the Rhinopithecus bieti fecal microbiome reveals a broad diversity of bacterial and glycoside hydrolase profiles related to lignocellulose degradation
Source: BMC Genomics. 2015 Mar 12;16(1):174. doi: 10.1186/s12864-015-1378-7 (PMC4369366; doi:10.1186/s12864-015-1378-7)
Supplement: Additional file 5: — Phylogenetic classification of viruses in the R. bieti metagenome. [file 12864_2015_1378_MOESM5_ESM.docx]

**Additional file 5 Phylogenetic classification of viruses in the *R. bieti* metagenome**

| phylum | order | family | species | JSH* (%) |
| --- | --- | --- | --- | --- |
| dsDNA viruses, no RNA stage | Caudovirales | Myoviridae | Enterobacteria phage PsP3 | 0.001 |
|  |  |  | Enterococcus phage phiEF24C | 0.001 |
|  |  |  | Listeria phage A511 | 0.001 |
|  |  |  | Vibrio phage nt-1 sensu lato | 0.001 |
|  |  |  | Bacillus phage 0305phi8-36 | 0.002 |
|  |  |  | Brochothrix phage A9 | 0.001 |
|  |  |  | Campylobacter phage CP220 | 0.001 |
|  |  |  | Clostridium phage c-st | 0.003 |
|  |  |  | Clostridium phage phiC2 | 0.002 |
|  |  |  | Escherichia phage rv5 | 0.001 |
|  |  |  | Lactobacillus phage LBR48 | 0.001 |
|  |  |  | Streptococcus phage EJ-1 | 0.001 |
|  |  | Podoviridae | Pseudomonas phage LUZ24 | 0.001 |
|  |  |  | Pseudomonas phage PaP3 | 0.001 |
|  |  |  | Enterobacteria phage N4 | 0.001 |
|  |  |  | Pseudomonas phage LUZ7 | 0.002 |
|  |  |  | Salmonella phage HK620 | 0.001 |
|  |  |  | Ralstonia phage RSB1 | 0.001 |
|  |  |  | Actinomyces phage Av-1 | 0.001 |
|  |  |  | Burkholderia phage Bcep22 | 0.001 |
|  |  |  | Myxococcus phage Mx8 | 0.001 |
|  |  | Siphoviridae | Pseudomonas phage MP22 | 0.001 |
|  |  |  | Bacillus phage SPbeta | 0.006 |
|  |  |  | Bacillus phage BCJA1c | 0.002 |
|  |  |  | Clostridium phage phi3626 | 0.002 |
|  |  |  | Clostridium phage phiCD6356 | 0.001 |
|  |  |  | Clostridium phage phiCP39-O | 0.002 |
|  |  |  | Clostridium phage phiCTP1 | 0.001 |
|  |  |  | Enterococcus phage EFAP-1 | 0.001 |
|  |  |  | Enterococcus phage EFRM31 | 0.001 |
|  |  |  | Enterococcus phage phiEf11 | 0.001 |
|  |  |  | Enterococcus phage phiFL2A | 0.001 |
|  |  |  | Enterococcus phage phiFL4A | 0.002 |
|  |  |  | Escherichia phage K1-dep(4) | 0.001 |
|  |  |  | Lactobacillus prophage Lj965 | 0.001 |
|  |  |  | Lactococcus phage 1706 | 0.003 |
|  |  |  | Lactococcus phage 936 sensu lato | 0.001 |
|  |  |  | Lactococcus phage TP901-1 | 0.001 |
|  |  |  | Lactococcus phage Tuc2009 | 0.001 |
|  |  |  | Lactococcus phage bIL285 | 0.005 |
|  |  |  | Lactococcus phage r1t | 0.002 |
|  |  |  | Rhodococcus phage ReqiDocB7 | 0.002 |
|  |  |  | Rhodococcus phage ReqiPepy6 | 0.008 |
|  |  |  | Rhodococcus phage ReqiPoco6 | 0.005 |
|  |  |  | Salmonella phage SETP12 | 0.001 |
|  |  |  | Salmonella phage SETP3 | 0.002 |
|  |  |  | Salmonella phage SETP5 | 0.001 |
|  |  |  | Staphylococcus phage 53 sensu lato | 0.001 |
|  |  |  | Staphylococcus phage phiETA3 | 0.001 |
|  |  |  | Streptococcus phage 2972 | 0.001 |
|  |  |  | Streptococcus phage Dp-1 | 0.002 |
|  |  |  | Streptococcus phage MM1 | 0.002 |
|  |  |  | Streptococcus phage PH10 | 0.002 |
|  |  |  | Streptococcus phage SM1 | 0.002 |
|  |  |  | Streptococcus phage phi3396 | 0.001 |
|  |  |  | Temperate phage phiNIH1.1 | 0.003 |
|  |  | unclassified (derived from Caudovirales) | Bacillus virus 1 | 0.002 |
|  |  | unclassified (derived from Caudovirales) | Geobacillus phage GBSV1 | 0.002 |
|  | unclassified (derived from Viruses) | Mimiviridae | Acanthamoeba polyphaga mimivirus | 0.006 |
|  | unclassified (derived from Viruses) | Phycodnaviridae | Micromonas sp. RCC1109 virus MpV1 | 0.001 |
|  | unclassified (derived from Viruses) | Poxviridae | Amsacta moorei entomopoxvirus 'L' | 0.001 |
|  | unclassified (derived from Viruses) | Retroviridae | Feline leukemia virus | 0.001 |
|  | unclassified (derived from Viruses) | Retroviridae | Human endogenous retrovirus | 0.001 |
|  | unclassified (derived from Viruses) | unclassified (derived from Viruses) | Abalone shriveling syndrome-associated virus | 0.001 |
|  | unclassified (derived from Viruses) | unclassified (derived from Viruses) | Bacillus phage 11143 | 0.001 |
|  | unclassified (derived from Viruses) | unclassified (derived from Viruses) | Bacillus phage lambda Ba01 | 0.001 |
|  | unclassified (derived from Viruses) | unclassified (derived from Viruses) | Bacillus phage phBC6A51 | 0.001 |
|  | unclassified (derived from Viruses) | unclassified (derived from Viruses) | Bacillus phage phBC6A52 | 0.001 |
|  | unclassified (derived from Viruses) | unclassified (derived from Viruses) | Lactobacillus phage Sal1 | 0.002 |
|  | unclassified (derived from Viruses) | unclassified (derived from Viruses) | Lactococcus phage | 0.002 |
|  | unclassified (derived from Viruses) | unclassified (derived from Viruses) | Phage Gifsy-1 | 0.001 |
|  | unclassified (derived from Viruses) | unclassified (derived from Viruses) | Phage Gifsy-2 | 0.001 |
|  | unclassified (derived from Viruses) | unclassified (derived from Viruses) | Staphylococcus phage P954 | 0.001 |
|  | unclassified (derived from Viruses) | unclassified (derived from Viruses) | Staphylococcus phage ROSA | 0.001 |
|  | unclassified (derived from Viruses) | unclassified (derived from Viruses) | Streptococcus phage 2096.1 | 0.002 |
|  | unclassified (derived from Viruses) | unclassified (derived from Viruses) | Streptococcus phage 370.1 | 0.003 |
|  | unclassified (derived from Viruses) | unclassified (derived from Viruses) | Streptococcus pyogenes phage 315.1 | 0.001 |
|  | unclassified (derived from Viruses) | unclassified (derived from Viruses) | Streptococcus pyogenes phage 315.4 | 0.003 |
|  | unclassified (derived from Viruses) | unclassified (derived from Viruses) | uncultured phage | 0.002 |
|  | unclassified (derived from Viruses) | unclassified (derived from Viruses) | uncultured phage MedDCM-OCT-S04-C64 | 0.001 |

* Percentage of sequences identified in metagenome of *R bieti*.
