# Supplementary figures and images for: Metagenomic analysis of the Rhinopithecus bieti fecal microbiome reveals a broad diversity of bacterial and glycoside hydrolase profiles related to lignocellulose degradation
Source: BMC Genomics. 2015 Mar 12;16(1):174. doi: 10.1186/s12864-015-1378-7 (PMC4369366; doi:10.1186/s12864-015-1378-7)

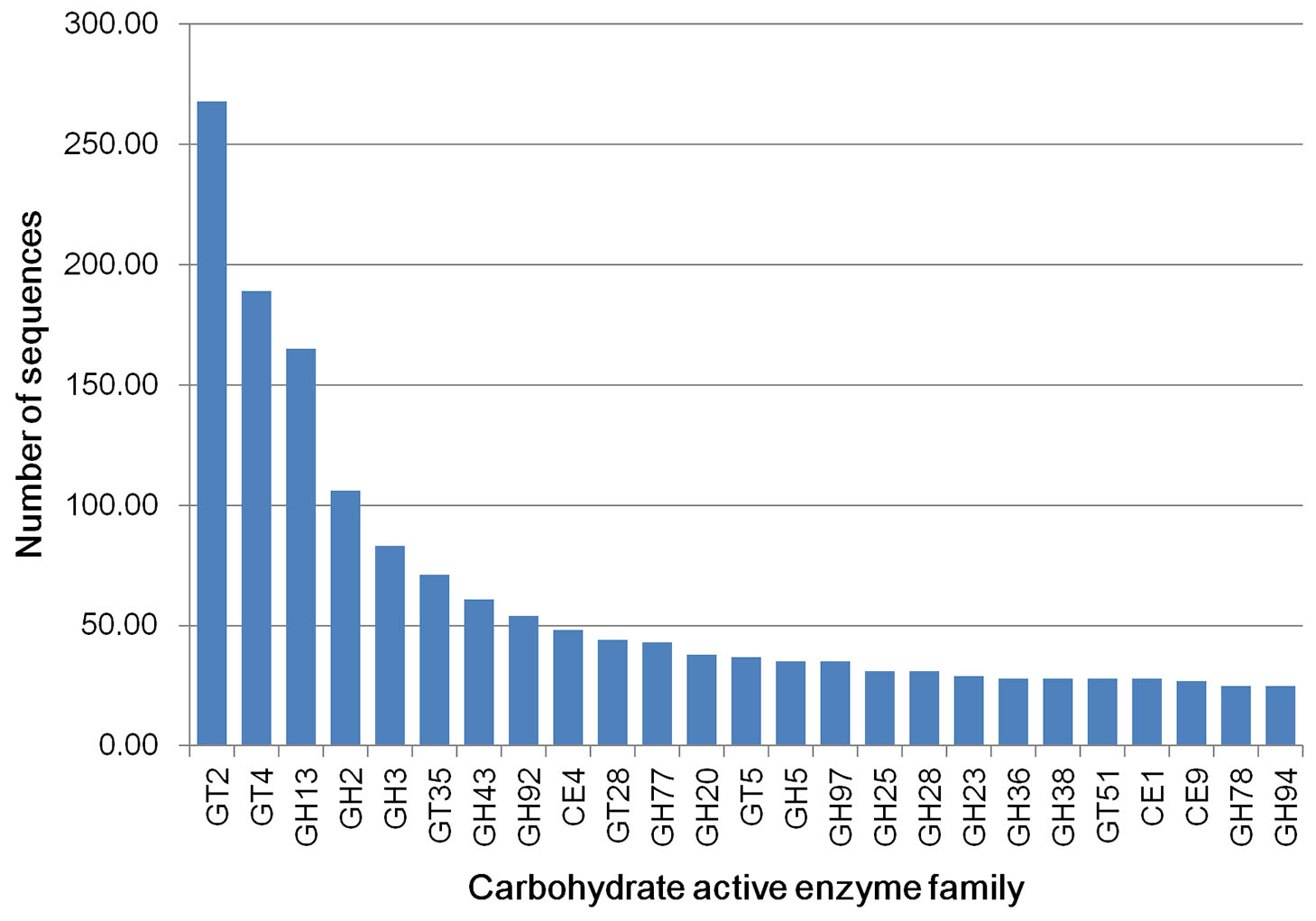

Supplement: Additional file 6: — Number of occurrences for the 25 most dominant carbohydrate active enzyme families. In total, these families contain 1557 sequences, representing 67.3% of the putative carbohydrate active enzyme family. [file 12864_2015_1378_MOESM6_ESM.jpeg]

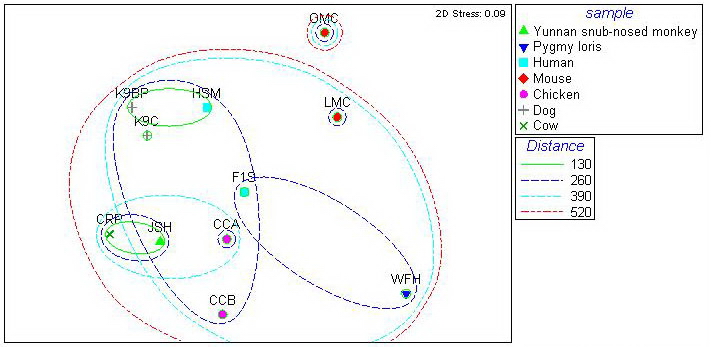

Supplement: Additional file 10: — Non-metric multidimensional scaling analysis based on relative abundance of taxonomic profiles among R. bieti , pygmy loris, human, mouse, canine, cow, and chicken gastrointestinal metagenomes. Samples of the same host species are indicated by the same symbol. Superimposed circles represent clusters of samples at different distance values (Euclidean distance). [file 12864_2015_1378_MOESM10_ESM.jpeg]

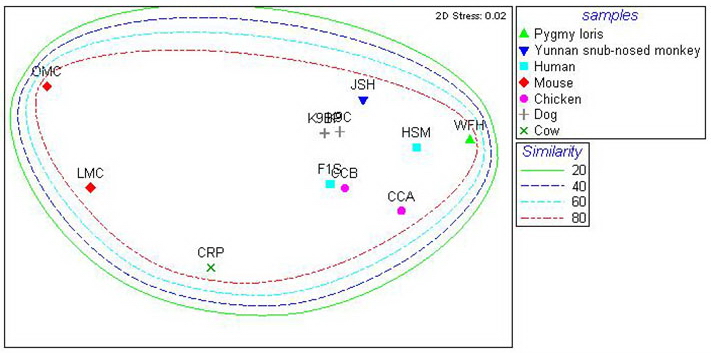

Supplement: Additional file 11: — Non-metric multidimensional scaling analysis based on relative abundance of metabolic profiles among R. bieti , pygmy loris, human, mouse, canine, cow, and chicken gastrointestinal metagenomes. Samples of the same host species are indicated by the same symbol. Superimposed circles represent clusters of samples at different similarity values (Bray-Curtis similarity). [file 12864_2015_1378_MOESM11_ESM.jpeg]
