# Supplementary material for: Metagenomic analysis of the Rhinopithecus bieti fecal microbiome reveals a broad diversity of bacterial and glycoside hydrolase profiles related to lignocellulose degradation
Source: BMC Genomics. 2015 Mar 12;16(1):174. doi: 10.1186/s12864-015-1378-7 (PMC4369366; doi:10.1186/s12864-015-1378-7)
Supplement: Additional file 7: — Presence of carbohydrate active enzyme families in the R. bieti metagenome. [file 12864_2015_1378_MOESM7_ESM.pdf]

Additional file 7 Presence of carbohydrate active enzyme families in the *R. bieti* metagenome

GH family*	Sequence #	% of total GH	GT family*	Sequence #	% of total GT
GH1	12	0.92%	GT1	7	0.94%
GH2	106	8.11%	GT2	268	36.12%
GH3	83	6.35%	GT3	6	0.81%
GH4	14	1.07%	GT4	189	25.47%
GH5	35	2.68%	GT5	37	4.99%
GH8	1	0.08%	GT8	14	1.89%
GH9	12	0.92%	GT10	1	0.13%
GH10	13	0.99%	GT11	1	0.13%
GH11	1	0.08%	GT14	4	0.54%
GH13	165	12.62%	GT19	9	1.21%
GH16	21	1.61%	GT20	3	0.40%
GH17	2	0.15%	GT25	3	0.40%
GH18	19	1.45%	GT26	10	1.35%
GH19	1	0.08%	GT28	44	5.93%
GH20	38	2.91%	GT30	9	1.21%
GH23	29	2.22%	GT32	10	1.35%
GH24	10	0.77%	GT35	71	9.57%
GH25	31	2.37%	GT39	7	0.94%
GH26	9	0.69%	GT41	2	0.27%
GH27	9	0.69%	GT50	1	0.13%
GH28	31	2.37%	GT51	28	3.77%
GH29	24	1.84%	GT55	1	0.13%
GH30	15	1.15%	GT56	1	0.13%
GH31	20	1.53%	GT66	1	0.13%
GH32	6	0.46%	GT70	1	0.13%
GH33	15	1.15%	GT82	1	0.13%
GH35	18	1.38%	GT83	6	0.81%
GH36	28	2.14%	GT84	7	0.94%
GH37	2	0.15%	**Totle GT**	**742**	**100.00%**
GH38	28	2.14%			
GH39	4	0.31%	**CE family***	**Sequence #**	**% of total CE**
GH42	13	0.99%	CE1	28	18.42%
GH43	61	4.67%	CE2	6	3.95%
GH45	1	0.08%	CE3	2	1.32%
GH50	3	0.23%	CE4	48	31.58%
GH51	12	0.92%	CE7	4	2.63%
GH53	8	0.61%	CE8	9	5.92%
GH55	4	0.31%	CE9	27	17.76%
GH57	16	1.22%	CE11	8	5.26%
GH59	1	0.08%	CE12	7	4.61%
GH63	6	0.46%	CE14	9	5.92%

GH64	3	0.23%	CE15	4	2.63%
GH65	11	0.84%	**Totle CE**	**152**	**100.00%**
GH67	1	0.08%			
GH73	19	1.45%	**PL family***	**Sequence #**	**% of total PL**
GH74	1	0.08%	PL1	6	21.43%
GH76	6	0.46%	PL6	1	3.57%
GH77	43	3.29%	PL9	1	3.57%
GH78	25	1.91%	PL10	5	17.86%
GH79	1	0.08%	PL11	9	32.14%
GH81	5	0.38%	PL12	1	3.57%
GH84	1	0.08%	PL15	2	7.14%
GH85	2	0.15%	PL17	3	10.71%
GH87	1	0.08%	**Totle PL**	**28**	**100.00%**
GH88	7	0.54%			
GH89	7	0.54%	**CBM family***	**Sequence #**	**% of total CBM**
GH92	54	4.13%	CBM3	2	2.33%
GH94	25	1.91%	CBM4	9	10.47%
GH95	19	1.45%	CBM6	6	6.98%
GH97	35	2.68%	CBM9	3	3.49%
GH98	1	0.08%	CBM12	3	3.49%
GH102	1	0.08%	CBM13	5	5.81%
GH103	2	0.15%	CBM16	3	3.49%
GH105	18	1.38%	CBM26	1	1.16%
GH106	18	1.38%	CBM30	1	1.16%
GH108	1	0.08%	CBM32	14	16.28%
GH109	10	0.77%	CBM33	1	1.16%
GH110	5	0.38%	CBM34	3	3.49%
GH111	2	0.15%	CBM35	2	2.33%
GH112	1	0.08%	CBM41	3	3.49%
GH115	11	0.84%	CBM48	12	13.95%
GH116	1	0.08%	CBM50	7	8.14%
GH120	4	0.31%	CBM51	2	2.33%
GH123	3	0.23%	CBM54	1	1.16%
GH125	9	0.69%	CBM57	3	3.49%
GH126	1	0.08%	CBM61	1	1.16%
GH127	12	0.92%	CBM62	4	4.65%
GH128	2	0.15%	**Totle CBM**	**86**	**100.00%**
GH130	12	0.92%			
**Totle GH**	**1307**	**100.00%**			

* Based on CAZy database (www.cazy.org)
