# Supplementary material for: Metagenomic analysis of the Rhinopithecus bieti fecal microbiome reveals a broad diversity of bacterial and glycoside hydrolase profiles related to lignocellulose degradation
Source: BMC Genomics. 2015 Mar 12;16(1):174. doi: 10.1186/s12864-015-1378-7 (PMC4369366; doi:10.1186/s12864-015-1378-7)
Supplement: Additional file 8: — GH profiles targeting plant structural polysaccharides in the R. bieti and other metagenomes. [file 12864_2015_1378_MOESM8_ESM.docx]

**Additional file 8** GH profiles targeting plant structural polysaccharides in the R. bieti and other metagenomes

| CAZy family* | Human[10] | Macropod [14] | Termite[11] | Cow rumen [15] | *R. bieti* |
| --- | --- | --- | --- | --- | --- |
| Cellulases |  |  |  |  |  |
| GH5 | 155 | 10 | 56 | 1451 | 35 |
| GH6 | 0 | 0 | 0 | 0 | 0 |
| GH7 | 0 | 0 | 0 | 1 | 0 |
| GH9 | 1 | 0 | 9 | 795 | 12 |
| GH44 | 0 | 0 | 6 | 0 | 0 |
| GH45 | 0 | 0 | 4 | 115 | 1 |
| GH48 | 0 | 0 | 0 | 3 | 0 |
| Total | **156(3)** | **10(2)** | **75(11)** | **2365(9)** | **48(4)** |
| Endohemicellulases | | | | | |
| GH8 | 48 | 1 | 5 | 329 | 1 |
| GH10 | 86 | 11 | 46 | 1025 | 13 |
| GH11 | 0 | 0 | 14 | 165 | 1 |
| GH12 | 0 | 0 | 0 | 0 | 0 |
| GH26 | 104 | 5 | 15 | 369 | 9 |
| GH28 | 10 | 2 | 6 | 472 | 31 |
| GH53 | 0 | 9 | 12 | 0 | 8 |
| Total | **248(4)** | **28(5)** | **98(14)** | **2360(9)** | **63(5)** |
| Debranching enzymes | | | | | |
| GH51 | 0 | 12 | 18 | 0 | 12 |
| GH54 | 0 | 0 | 0 | 0 | 0 |
| GH62 | 0 | 0 | 0 | 1 | 0 |
| GH67 | 60 | 5 | 10 | 120 | 1 |
| GH78 | 0 | 25 | 0 | 1260 | 25 |
| Total | **60(1)** | **42(8)** | **28(4)** | **1381(5)** | **38(3)** |
| Oligosacchride-degrading enzymes | | | | | |
| GH1 | 229 | 61 | 22 | 253 | 12 |
| GH2 | 94 | 24 | 23 | 1436 | 106 |
| GH3 | 1102 | 72 | 69 | 2844 | 83 |
| GH29 | 386 | 2 | 0 | 939 | 24 |
| GH35 | 97 | 3 | 3 | 158 | 18 |
| GH38 | 222 | 3 | 11 | 272 | 28 |
| GH39 | 39 | 1 | 3 | 315 | 4 |
| GH42 | 135 | 8 | 24 | 374 | 13 |
| GH43 | 0 | 10 | 16 | 0 | 61 |
| GH52 | 0 | 0 | 3 | 0 | 0 |
| Total | **2304(37)** | **184(33)** | **174(25)** | **6591(24)** | **349(27)** |
| Total GHs identified | **6188** | **557** | **704** | **27755** | **1307** |

* Based on CAZy database (www.cazy.org).

The numbers in parentheses represent the percentages of these groups relative to the total number of GH’s identified in the metagenomic datasets.
