# Supplementary material for: Metagenomic analysis of the Rhinopithecus bieti fecal microbiome reveals a broad diversity of bacterial and glycoside hydrolase profiles related to lignocellulose degradation
Source: BMC Genomics. 2015 Mar 12;16(1):174. doi: 10.1186/s12864-015-1378-7 (PMC4369366; doi:10.1186/s12864-015-1378-7)
Supplement: Additional file 9: — Overview of the MG-RAST metagenomes chosen for comparison. [file 12864_2015_1378_MOESM9_ESM.pdf]

Additional file 9 Overview of the MG-RAST metagenomes chosen for comparison

Metagenome (MG-RAST accession)	Host	Sequence number	Total Size MB	Shortest	Longest	Average
JSH (4452795.3)	*R. bieti*	88514	35.1	100	700	396
LMC (4440463.3)	Lean mouse	10845	8.4	77	1307	781.8
OMC (4440464.3)	Obese mouse	11857	9.1	112	1187	764.7
F1S (4440939.3)	Human	28900	38	92	16490	1315
HSM (4444130.3)	Malnourished Human	108486	74.2	93	160132	684
CCA (4440283.3)	Chicken	310801	32.3	39	258	103
CCB (4440284.3)	Chicken	254712	26.4	40	249	103
K9C (4444164.3)	Dog (low-fiber diet)	66969	53.2	44	36188	794
K9BP (4444165.3)	Dog (beet pulp diet)	67761	43.6	41	14401	642
CRP (4441682.3)	Cow	218460	22.5	35	193	102
WFH (4476304.3)	Pygmy loris	61281	27.2	49	1201	443
